# Association of Single Nucleotide Polymorphisms in Estrogen Receptor Alpha Gene with Susceptibility to Knee Osteoarthritis: A Case-Control Study in a Chinese Han Population

**DOI:** 10.1155/2014/151457

**Published:** 2014-03-17

**Authors:** Xiaoyu Dai, Chao Wang, Jin Dai, Dongquan Shi, Zhihong Xu, Dongyang Chen, Huajian Teng, Qing Jiang

**Affiliations:** ^1^The Center of Diagnosis and Treatment for Joint Disease, Drum Tower Hospital Affiliated to Medical School of Nanjing University, Zhongshan Road 321, Nanjing, Jiangsu 210008, China; ^2^Joint Research Center for Bone and Joint Disease, Model Animal Research Center (MARC), Nanjing University, Nanjing, Jiangsu 210093, China

## Abstract

Osteoarthritis (OA) is the most prevalent form of arthritis and its multifactorial nature has been increasingly recognized. Genetic factors play an important role in OA etiology and estrogen receptor alpha (ESR1) gene polymorphisms may be involved. This study tried to explore whether the ESR1 gene single nucleotide polymorphisms (SNPs) were associated with primary knee OA in the Chinese Han population. Two SNPs, rs2234693 and rs9340799, were genotyped in 469 cases and 522 controls. Rs2234693 was associated with knee OA in the dominant genetic model (TT + TC versus CC) (*P* = 0.025) and a higher T allele frequency existed (*P* = 0.047) among females. The combined genotype (TT + TC) (*P* = 0.025) and T allele (*P* = 0.016) were related with mild knee OA only. For rs9340799, A allele was associated with knee OA in all subjects (*P* = 0.031) and females (*P* = 0.046). Statistical differences were detected in the dominant genetic model (AA + AG versus GG) among females (*P* = 0.030). The combined genotype (AA + AG) (*P* = 0.036) and A allele (*P* = 0.039) were merely correlated with mild knee OA. ESR1 gene is considerably associated with knee OA etiology in the Chinese Han population.

## 1. Introduction

Osteoarthritis (OA, OMIM#165720) is a multifactorial disorder characterized by progressive cartilage loss, osteophyte formation, and subchondral sclerosis, which accounts for a large amount of elderly individuals with pain and disability [1, 2]. Joint arthroplasty surgery, mostly at the knee and the hip, acts as the major therapeutic method for severe OA in current times despite its invasive nature and a large economic burden [3]. In this regard, a better understanding of the etiology of OA is much required for a more effective and targeted prevention.

To date, several large-sample studies and genome-wide association studies (GWAS) have shed further light on potential chromosome regions that may carry OA susceptibility genes which have been subsequently confirmed by replication studies in different populations [4–6]. Asia population have an especially high incidence of knee OA and a recent study has also indicated that the prevalence of symptomatic and radiographic knee OA in Chinese females aged 60 and over was 15.4% and 42.8%, respectively [7]. Combined with the observation that women were more vulnerable to more severe knee OA especially after menopausal age [8], it could be hypothesized that estrogen may be involved in the onset or progression of knee OA.

The human estrogen receptor has two isoforms: ESR1 and ESR2, which are members of the steroid/thyroid hormone superfamily of nuclear receptors and encoded by separate genes [9, 10]. ESR1 is expressed in chondrocytes, stromal cells, and osteoblasts [11], which potentially indicated that both bone and cartilage can be regulated by ESR1 gene. Genetically, there have been several studies on associations between two common ESR1 gene polymorphisms (rs2234693 and rs9340799) and OA, and the findings were controversial [12]. A considerable number of published reports have indicated a significant association of ESR1 gene polymorphisms and risk for knee, hip, or generalized OA in different populations, although the potential mechanisms await clarifications [13–19]. Still, some investigators failed to demonstrate the possible effects of ESR1 gene polymorphisms on knee and hip OA [20]. For the first time, this study is an attempt to examine the genetic association of the ESR1 gene polymorphism (rs2234693 and rs9340799) with knee OA in the Chinese Han population.

## 2. Materials and Methods

### 2.1. Subjects

A total of 991 subjects were enrolled in this study. 469 patients with primary knee OA (357 females and 113 males) were recruited consecutively at the Center of Diagnosis and Treatment for Joint Disease, Drum Tower Hospital, affiliated to the Medical School of Nanjing University, and 522 age-matched healthy controls (124 females and 398 males) were consecutively selected in a plenty of more than 2000 individuals at the Center of Physical Examination. All subjects included in this study were Han Chinese living in and around Nanjing and no one dropped out. The study was approved by the ethics committee of the Medical School of Nanjing University, and informed consent was obtained from patients and control participants.

The inclusion criteria for both OA patients and controls were previously described by Jiang et al. [21]. Informationregardingthe health conditions which mainly include hypertension, hyperglycemia or diabetes, and hyperlipidemia were collected according to their self-report due to the potential confounding effects exerted on knee OA by these disease conditions [1]. Notably, considering the significant associations between ESR1 gene polymorphisms and hypertension in several case-control studies [22–24], patients with hypertension failed to be included. There were 11 (2.35%) and 19 (3.64%) patients who had hyperglycemia or diabetes in case and control groups, respectively. No patients had hyperglycemia. We then calculated the body mass index as weight (kg) divided by height squared (m^2^) for all the participants to estimate obesity roughly. All the knee OA patients had not only definite symptoms and signs (pain, tenderness, swelling, or restricted motion) of OA for at least 5-month duration but also radiographic evidences which were diagnosed by standardized anteroposterior radiographs of the knee joint in extension. For each patient, the Kellgren/Lawrence (K/L) grading system was adopted to evaluate radiographic OA [25], and only patients with K/L grades of 2 or higher were included. We then classified radiographic OA findings into mild OA (K/L grade 2) and severe OA (K/L grade 3 or 4) for a clear definition of the severity. Exclusion criteria for this study include other inflammatory arthritis (rheumatoid, autoimmune, or polyarthritic disease), posttraumatic or postseptic arthritis, and developmental dysplasia or skeletal dysplasia.

### 2.2. Genotyping

The minor allele frequencies of both rs2234693 and rs9340799 were above 0.2 in the Chinese population, respectively. DNA samples were obtained from all the participants from peripheral blood adopting the Chelex-100 method [26] or buccal swabs using the DNA IQ System (Promega, Madison, WI) based on the manufacturer's instructions. The two SNPs were then genotyped using Taqman assay (Applied Biosystems 7500, ABI, Foster City, CA). Genotyping was performed by laboratory personnel who were blinded to case status, and three authors independently reviewed the genotyping results, data entry, and statistical analyses. In addition, we randomly selected 5% samples of case and control subjects for reproducibility tests at least twice in different days and yielded a 100% concordant.

### 2.3. Statistic Analysis

Mean values were presented with their standard deviation (SD) and assessed by Students' *t*-test and standard *χ* ^2^ tests were employed to determine the significance of differences in allelic and genotypic distributions between cases and controls. Multivariate logistic regression was used to estimate odds ratios (ORs) and 95% confidence intervals (CI) after adjustment for age and BMI for estimating the associations between the SNPs and the risk of knee OA. Stratifications for gender and the severity of OA were subsequently conducted for further analyses. The Hardy-Weinberg Equilibrium (H-WE) was tested by a goodness-of-fit *χ* ^2^ test to compare the allele and genotype proportions in case and control subjects. For all the tests, a two-tailed probability value of less than 0.05 was considered as statistically significant. All the data was analysed by SPSS 19.0 (IBM SPSS, Chicago, USA).

## 3. Results

Baseline characteristics of all the subjects were shown in Table 1. The mean age of knee OA patients (57.3 ± 10.9) and controls (56.4 ± 9.8) are not significantly different (*P* = 0.473). It could be noted that knee OA patients had a higher mean BMI than that of control subjects (*P* < 0.001). Approximately 45.6% of the OA patients had a K/L score of 3 or 4. However, the mismatching in sex distribution between two groups existed in this study (*P* < 0.001). Tables 2 and 3 showed the genotyped and allele distributions for cases and controls based on the stratification of gender and K/L grades. Distributions of genotypes and alleles of two SNPs in healthy control individuals were all confirmed to Hardy-Weinberg Equilibrium (0.997 and 0.736, resp.). The minor allele frequencies of rs2234693 (allele C) and rs9340799 (allele G) in control subjects were 0.379 and 0.185, respectively, which were close to those reported in HapMap for the Chinese Han population (0.402 and 0.211, resp.).

In the association study, for rs2234693, we did not observe any significant difference in any comparison as a whole (Table 4). When stratified by gender after adjustment for age and BMI, we found evident differences in the dominant model (TT + TC versus CC) in female subjects (OR = 2.087; 95% CI, 1.086–4.009; *P* = 0.025). In addition, a higher T allele frequency was also associated with an increased risk of knee OA in females (OR = 1.359; 95% CI, 1.003–1.840; *P* = 0.047). For males, no differences were found to be statistically significant. After stratification for the severity of OA, apparent differences were detected in the comparison of CC versus other genotyped combined (TT + TC) after adjustment for age, BMI, and sex in patients with mild knee OA (OR = 1.560; 95% CI, 1.056–2.304; *P* = 0.025) (Table 6). Still, T allele was modestly related with an elevated risk of mild knee OA (OR = 1.320; 95% CI, 1.050–1.615; *P* = 0.016). Unexpectedly, we failed to find any relationship between genotype or allele frequencies of rs2234693 and the risk of severe knee OA.

With respect to rs9340799, A allele was relevant to a higher risk of knee OA in all the patients and controls (OR = 1.272; 95% CI, 1.022–1.583; *P* = 0.031) and this difference remained in female subjects (OR = 1.454; 95% CI, 1.006–2.102; *P* = 0.046) (Table 5). The most significant difference in the dominant model (AA + AG versus GG) was also shown in female subjects (OR = 4.410; 95% CI, 1.027–18.938; *P* = 0.030). Similarly, the combined genotype (AA + AG) (OR = 2.011; 95% CI, 1.036–3.902; *P* = 0.036) and A allele (OR = 1.312; 95% CI, 1.013–1.701; *P* = 0.039) were merely correlated with the elevated risk of mild knee OA (Table 7).

## 4. Discussion

To our best knowledge, this case-control study firstly described a compelling association of ESR1 SNPs (rs2234693 and rs9340799) and primary knee OA in a Chinese Han population with a complete exclusion of those who had hypertension previously or currently. Significant differences were found between 469 patients and 522 control subjects in the two SNPs. Summarily, we herein suggested the leading roles of T allele of rs2234693 and A allele of rs9340799 in the pathogenesis of primary knee OA, especially for female patients and those subjects with mild disease. For these patients, a potential individualized prevention and treatment of knee OA may be practicable.

Until now, a relative lack of consistency or reproducibility of the association between ESR1 gene (rs2234693 and rs9340799) polymorphisms and OA still exists. Previously, Ushiyama et al. [13] reported an increased risk of generalized OA with genotypes of rs2234693 and rs9340799 polymorphisms in 383 Japanese women. In a large-scale population-based study of Caucasians, Bergink et al. [14] found that ESR1 haplotypes of rs2234693 and rs9340799 polymorphisms were significantly relevant to radiographic knee OA, especially for those who had osteophytes with adjustment for bone mineral density (BMD). Also, one study showed no correlation between ESR1 gene polymorphisms and idiopathic OA of the knee and the hip in a Caucasian population (371 OA patients who underwent total joint replacement and 369 controls) [20]. Recently, ESR1 gene haplotype had been confirmed to be associated with primary knee OA in Korean and Mexican mestizo populations [15, 19]. After accounting for femoral neck BMD, a decreased prevalence of hip OA phenotype which was characterized by moderate to severe joint space narrowing in presence of CC genotype in SNP rs2234693 was also reported in Caucasians [17]. Still, associations of the CC genotype with a reduced risk of knee OA in women and an increased risk of hip OA in men were found in Europeans [18].

Noteworthy, the C allele frequency of rs2234693 and the G allele frequency of rs9340799 in controls were 0.379 and 0.185, respectively, which were similar to those observed in the Japanese and Korean populations [13, 15] whereas different from those in European and American Caucasians to a certain degree [17, 18]. We also proposed the predisposing roles of T allele of rs2234693 in the etiology of knee OA, and this was partially in concordance with the protective effects of C allele in Japanese and Caucasians [17, 18] but against the observation that CC genotype was a risk factor of radiographic knee OA in a Rotterdam study [14]. Still, this difference itself may indicate the heterogeneity in association with specific genetic polymorphisms in different ethnic groups. Environment factors such as anatomical and biomechanical effects and some joint-specific hereditary factors may also be involved in the influencing process on OA susceptibility by certain polymorphic locus [27, 28]. Likewise, potential variations in the nature of OA genetic susceptibility in different joints seem to be a reasonable interpretation [27]. In addition, the different inclusion criteria in the aforementioned studies may also be a possible explanation for the discrepancy. Summarily, further studies on different anatomic OA after unifying the recruitment criteria and OA end points of subjects in various populations will be enormously beneficial to a better understanding of these polymorphisms in OA etiology.

The lack of difference in male participants in this study may be due to the limited sample number, despite a large sex bias of OA incidence in females [8]. With adjustment for age, BMI, and gender, we found that both of the rs2234693 and rs9340799 were in relation with the risk of mild knee OA, whereas the associations disappeared in patients with severe knee OA, which indicated that the SNPs might play a certain role in the progress of OA at different stages. The findings were consistent with that of an Oxford study in patients who accepted joint arthroplasty due to severe OA of the lower limb [20] but against the results reported by Jin et al. in Koreans [15]. The analysis of different OA end points used in different studies may be an explanation. Hence a definite conclusion cannot be made and more large-scale population studies will be much required to clarify this finding. Still, we cannot exclude the possibility that the two SNPs may be in linkage disequilibrium with some other more relevant alleles within the ESR1 gene region. Previous studies of candidate genes for OA susceptibility were mainly related to genes encoding collagens, extracellular matrix molecules, bone and cartilage growing factors, and the inflammation pathway [29, 30]. Thus, polygenic effects on the pathogenesis of knee OA should not be ignored. There still exists a necessity to explore other potentially contributing genes involved in the development of knee OA for a better comprehension of genetic regulation on knee OA.

Estrogens act on the skeleton through the binding to ESRs (ESR1 and ESR2), and ESR1 has been increasingly deemed as a mediator of considerable importance in the signal transduction pathway [10]. Given that rs2234693 and rs9340799 are located in intron 1, there is a possibility that TT and AA genotypes of rs2234693 and rs9340799 polymorphisms may exert influence on the expression of ESR1 in bone cells and chondrocytes through transcriptional process. The specific effects may be associated with changes in (juxta articular) bone or articular cartilage, subsequently leading to OA. The truth is that human ESR1 mRNA isoforms are synthesized by splicing of 1 of 6 alternative first exons (1A–1F) to a common acceptor site, which may lead to differential patterns of expression of ESR1 in different tissues and cells [31]. There also exist three isoforms of ESR1 of 36, 46, and 66 kDa, which were generated by alternative splicing of the gene and had certainly transcriptional and functional differences [32]. Previously, C allele of rs2234693 was found to produce a favorable and functional binding site for the* myb* transcription factor that can initiate a great promotion of transcription [33]; however, little has been known in terms of this potential influence on ESR1 expression up to now. On the other hand, Riancho et al. [18] recently pointed out that genotypes of rs2234693 were correlated with the statistically differences in CYP19A1 transcript abundance in a large-sample study, which might propose an underlying transinteraction of the two genes situated in different chromosomes. Accordingly, considering the fact that the expression pattern of ESR1 isoforms in the skeleton especially the cartilage remains unclear, further exploration concerning the impacts of ESR1 gene on OA pathogenesis from the perspective of transcriptional regulation will still be of critical importance.

OA has been seen as a chronic inflammatory disease which could affect the cartilage, the synovium, the subchondral bone, and the other joint tissues [34]. Much attention has been paid to the effects of estrogen on articular cartilage, and estrogen can also affect the synovium, muscles, ligaments, the preiarticular bone, and the joint capsule [35]. Conflicting results have been reported in experimental animal models in terms of the role of estrogen in the development of OA [36–38]. In clinic, several observational studies have shown the protective effects of estrogen replacement therapy (ERT) on OA, especially at the hip joint [39–41]. But, other published reports found short term of ERT may increase the risk of OA in the hip and hand [42, 43]. For all that, the fact that estrogen may have both anti- and proinflammatory properties depending on the involved joint tissues and the situation has been gradually accepted but the specific mechanisms are still unknown [44]. Therefore, more large-sample retrospective and prospective studies in terms of the duration of ERT in different joints will be much needed. By combining with the established association of polymorphisms in the ESR1 gene and OA susceptibility, it will also help to conduct a more resultful and targeted adjuvant treatment on these patients especially for postmenopausal women. Particularly, ERT should target the joint as a whole rather than focusing only on the damage of cartilage. A view that combined estrogen and progesterone therapy could reduce the severity of OA in a murine model of OA is likely to provide us a novel research direction again [45]. Estrogen has been found to decrease the levels of reactive oxygen species, inhibit the synthesis of interlukin-1*β*-mediated nitric oxide, and reduce the production of some proinflammation factors in articular cartilage [44, 46]. Getting insight into the mechanisms with which estrogen works on OA will also be conducive to a more comprehensive understanding of the genetic influence on OA by ESR1 gene.

Our study still has a few limitations. The relatively limited sample size and unmatched gender in patients and controls may influence the statistical power of any existing association. We failed to systematically obtain X-rays of the knee in control subjects to avoid the interference of asymptomatic OA. Also, we only evaluate the risk of knee OA and ESR1 gene polymorphisms, and the results cannot be well generalized to other joint sites. Nevertheless, there is reason to believe that the findings are of considerable credibility and veracity. Given the fact that genetic factors may vary with disease pattern and severity and according to individuals' characteristics such as gender and age [19], we still have a long way to go in the genetic research of OA.

## 5. Conclusions

In conclusion, we suggested an association of the polymorphisms of rs2234693 and rs9340799 in the ESR1 gene and susceptibility to primary knee OA in the Chinese Han population. This association remained in female patients and those who had mild OA. Future investigations should focus on sex-specific mechanisms on the etiology of knee OA and determine whether there are genetic factors which can be targeted through prevention and therapy strategies to mitigate the seemingly increased prevalence of knee OA.

## Figures and Tables

**Table 1:** Baseline characteristics of subjects.

	Cases	Controls	*P* value
Subjects, number	469	522	—
Females, number (%)	356 (75.9)	120 (23.0)	*P* < 0.001*
Mean age, y (SD)	57.3 ± 10.9	56.4 ± 9.8	*P* = 0.473
Mean BMI, kg/m^2 ^(SD)	26.1 ± 3.9	24.3 ± 2.8	*P* < 0.001
Kellgren-Lawrence grading			
Grade 2, number (%)	255 (54.5)	0 (0.0)	—
Grade 3, number (%)	119 (25.3)	0 (0.0)	—
Grade 4, number (%)	95 (20.2)	0 (0.0)	—

No.: number; y: years; SD: standard deviation.

*Pearson chi-square test.

**Table 2:** Genotype and allele frequencies of rs2234693 and rs9340799 of the ESR1 gene in the Han Chinese population.

Group	Number	rs2234693							rs9340799					
		Genotype (frequency)			Allele (frequency)		H-WE	Number	Genotype (frequency)			Allele (frequency)		H-WE
		TT	TC	CC	T	C	*P*		AA	AG	GG	A	G	*P*
OA														
All	469	167 (0.356)	217 (0.463)	85 (0.181)	551 (0.587)	387 (0.413)	0.325	469	288 (0.614)	152 (0.324)	29 (0.062)	728 (0.776)	210 (0.224)	0.144
Female	356	119 (0.334)	170 (0.478)	67 (0.188)	408 (0.573)	304 (0.427)	0.649	356	210 (0.590)	122 (0.343)	24 (0.067)	542 (0.761)	170 (0.239)	0.280
Male	113	48 (0.425)	47 (0.416)	18 (0.159)	143 (0.633)	83 (0.367)	0.264	113	77 (0.682)	30 (0.265)	6 (0.053)	184 (0.814)	42 (0.186)	0.192
Control														
All	514	198 (0.385)	242 (0.471)	74 (0.144)	638 (0.621)	390 (0.379)	0.997	522	348 (0.667)	155 (0.297)	19 (0.036)	851 (0.815)	193 (0.185)	0.736
Female	120	47 (0.392)	61 (0.508)	12 (0.100)	155 (0.646)	85 (0.0354)	0.223	124	82 (0.661)	40 (0.323)	2 (0.016)	204 (0.823)	44 (0.177)	0.242
Male	394	151 (0.383)	181 (0.459)	62 (0.158)	483 (0.613)	305 (0.387)	0.528	398	266 (0.668)	115 (0.289)	17 (0.043)	647 (0.813)	149 (0.187)	0.314

No.: number; H-WE: Hardy-Weinberg Equilibrium.

**Table 3:** Genotype and allele frequencies of rs2234693 and rs9340799 of the ESR1 gene with a stratification by K/L grades.

K/L grading	Number	rs2234693						rs9340799					
		Genotype (frequency)			Allele (frequency)		H-WE	Genotype (frequency)			Allele (frequency)		H-WE
		TT	TC	CC	T	C	*P* value	AA	AG	GG	A	G	*P* value
Grade 2	255	82 (0.321)	120 (0.471)	53 (0.208)	284 (0.557)	226 (0.443)	0.458	156 (0.612)	81 (0.318)	18 (0.070)	393 (0.771)	117 (0.229)	0.105
Grade 3	119	46 (0.387)	54 (0.454)	19 (0.159)	146 (0.613)	92 (0.387)	0.638	74 (0.622)	42 (0.353)	3 (0.025)	190 (0.798)	48 (0.202)	0.295
Grade 4	95	39 (0.410)	43 (0.453)	13 (0.137)	121 (0.637)	69 (0.363)	0.834	57 (0.600)	29 (0.305)	9 (0.095)	143 (0.753)	47 (0.247)	0.079

K/L: Kellgren-Lawrence; No.: number; H-WE: Hardy-Weinberg Equilibrium.

**Table 4:** Association of the rs2234693 with knee OA in the Chinese Han population with a stratification by gender.

Groups compared	TT versus TC + CC			TT + TC versus CC			T allele versus C allele			All genotyped*
	OR	95% CI	*P* value	OR	95% CI	*P* value	OR	95% CI	*P* value	*P* value
All patients (*n* = 469) versus all controls (*n* = 514)	1.133	0.874–1.469	0.345	1.316	0.937–1.850	0.113	1.149	0.959–1.377	0.133	0.259
Female patients (*n* = 356) and female controls (*n* = 120)	1.282	0.836–1.967	0.254	2.087	1.086–4.009	**0.025**	1.359	1.003–1.840	**0.047**	0.073
Male patients (*n* = 113) and male controls (394)	0.917	0.599–1.405	0.690	0.954	0.538–1.691	0.871	0.943	0.693–1.283	0.709	0.923

OR: Odds Ratio; CI: confidence interval.

*TT, TC, and CC genotypes were grouped together and a 2 × 3 contingency-table analysis was performed.

**Table 5:** Association of the rs9340799 with knee OA in the Chinese Han population with a stratification by gender.

Groups compared	AA versuss AG + GG			AA + AG versus GG			A allele versus G allele			All genotyped*
	OR	95% CI	*P* value	OR	95% CI	*P* value	OR	95% CI	*P* value	*P* value
All patients (*n* = 469) versus all controls (*n* = 522)	1.257	0.969–1.630	0.085	1.745	0.965–3.156	0.063	1.272	1.022–1.583	**0.031**	0.084
Female patients (*n* = 356) and female controls (*n* = 124)	1.357	0.885–2.082	0.161	4.410	1.027–18.938	**0.030**	1.454	1.006–2.102	**0.046**	0.069
Male patients (*n* = 113) and male controls (398)	0.801	0.510–1.256	0.332	1.125	0.433–2.925	0.809	0.869	0.594–1.271	0.468	0.817

OR: Odds Ratio; CI: confidence interval.

*AA, AG, and GG genotypes were grouped together and a 2 × 3 contingency-table analysis was performed.

**Table 6:** Association of the rs2234693 with knee OA in the Chinese Han population with a stratification by the severity of OA.

Groups compared	TT versus TC + CC			TT + TC versus CC			T allele versus C allele			All genotyped*
	OR	95% CI	*P* value	OR	95% CI	*P* value	OR	95% CI	*P* value	*P* value
Mild OA (255 patients versus 514 controls)	1.322	0.963–1.815	0.084	1.560	1.056–2.304	**0.025**	1.302	1.050–1.615	**0.016**	**0.047**
Severe OA (214 patients versus 514 controls)	0.951	0.686–1.318	0.763	1.045	0.667–1.638	0.846	0.986	0.782–1.245	0.908	0.910

OR: Odds Ratio; CI: confidence interval.

*TT, TC, and CC genotypes were grouped together and a 2 × 3 contingency-table analysis was performed.

**Table 7:** Association of the rs9340799 with knee OA in the Chinese Han population with stratification by the severity of OA.

Groups compared	AA versus AG + GG			AA + AG versus GG			A allele versus G allele			All genotype∗
	OR	95% CI	*P* value	OR	95% CI	*P* value	OR	95% CI	*P* value	*P* value
Mild OA (255 patients versus 522 controls)	1.269	0.930–1.732	0.132	2.011	1.036–3.902	**0.036**	1.312	1.013–1.701	**0.039**	0.073
Severe OA (214 patients versus 522 controls)	1.267	0.911–1.762	0.159	1.573	0.750–3.299	0.227	1.258	0.954–1.658	0.103	0.261

OR: Odds Ratio; CI: confidence interval.

*AA, AG, and GG genotypes were grouped together and a 2 × 3 contingency-table analysis was performed.
